# Role of platelet-rich plasma in unexplained recurrent implantation failure: an umbrella review

**DOI:** 10.3389/frph.2026.1856964

**Published:** 2026-07-08

**Authors:** Harpreet Kaur, Anil Chauhan, Muhammed Shabil, Mishu Mangla

**Affiliations:** 1 Department of Obstetrics and Gynaecology, All India Institute of Medical Sciences, Bilaspur, Himachal Pradesh, India; 2 Evidence-Based Health Informatics Unit, Regional Resource Centre, Department of Telemedicine, Post Graduate Institute of Medical Education & Research, Chandigarh, India; 3 Department of Pharmacy Practice, Faculty of Pharmacy, MS Ramaiah University of Applied Sciences, Bangalore, India; 4 Department of Obstetrics and Gynaecology, All India Institute of Medical Sciences, Bibinagar, Hyderabad, India

**Keywords:** ART, IVF, platelet-rich plasma (PRP), recurrent implantation failure, RIF, umbrella review

## Abstract

**Background/objective:**

The role of platelet-rich plasma (PRP) in assisted reproductive technology is being explored, particularly in cases of recurrent implantation failure (RIF). The objective of our study is to thoroughly synthesise and critically assess the available evidence from systematic reviews and meta-analyses regarding the use of PRP in women with unexplained RIF.

**Methods:**

A thorough web search was performed on Medline, PubMed, EMBASE, Web of Science, OVID, JBI Evidence Synthesis, the Cochrane Database of Systematic Reviews, DARE, and the PROSPERO register to identify systematic reviews addressing the research question. Systematic reviews were assessed based on their “explicit, reproducible methodology,” comprehensive search strategy, acceptable methods for assessing the validity of included studies, evaluation of the risk of bias, and assessment of the certainty of the evidence. The methodological quality was assessed with the AMSTAR tool comprising 11 items. Before compiling the results, a corrected covered area (CCA) metric was used to evaluate the degree of overlap. Data extraction included descriptive variables and details about the intervention and outcomes.

**Results:**

A total of 15 systematic reviews of randomised controlled trials are included. The CCA matrix revealed very high overlap (42%). Although our study showed an improvement in implantation rate, clinical pregnancy rate, and live birth rate, the certainty of evidence was either low or not reported. Therefore, it is difficult to make recommendations based on these findings as the quality of evidence was low.

**Discussion:**

Although the qualitative review of these reviews has shown a beneficial effect of PRP in RIF, this result cannot be recommended with certainty, as Grading of Recommendations, Assessment, Development, and Evaluation (GRADE) analysis was performed in only five of the 15 studies. Meta-analysis of the individual outcomes after extracting data from all studies also revealed an overall beneficial effect of PRP in terms of various outcomes, i.e., live birth rate, clinical pregnancy rate, and implantation. However, this may be an overestimation of the true effect due to the low quality of evidence or the quality of evidence not being assessed.

**Conclusion:**

Intrauterine infusion of PRP appears to be a promising adjunct therapy for women with unexplained recurrent implantation failure; however, the overall quality of evidence remains low.

**Systematic Review Registration:**

https://www.crd.york.ac.uk/PROSPERO/view/CRD42024504453, PROSPERO CRD42024504453.

## Graphical Abstract

**Figure:**
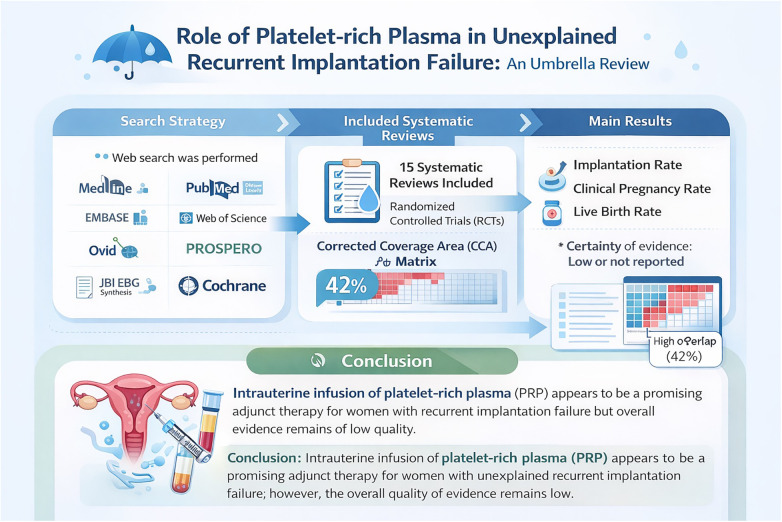
Role of platelet-rich plasma in unexplained recurrent implantation failure: an umbrella review.

## Introduction

The rapidly evolving field of assisted reproductive technology (ART) has allowed couples to achieve viable pregnancies, who previously could not conceive for a variety of reasons. One of the major challenges in ART is recurrent implantation failure (RIF) (1), which renders both the treating physician and couples frustrated and desperate. RIF refers to cases in which women have had at least three previous fresh or frozen embryo transfer (FET) failures. Several modalities have been explored to increase the pregnancy rate in ART cases. One such modality, which has been explored in recent years, is the use of platelet-rich plasma (PRP), particularly in cases of RIF. PRP, obtained from a person’s autologous blood samples, is several times richer in platelets and other growth factors, such as vascular endothelial growth factor (VEGF), transforming growth factor, platelet-derived growth factor (PDGF), and epidermal growth factor, than circulating blood (2). In recent years, the application of intrauterine infusion of autologous PRP to promote endometrial growth and receptivity has been increasing due to its positive effect on endometrial growth and pregnancy outcome (3). We encountered multiple systematic reviews and meta-analyses (4–6) on the use of PRP in RIF, showing the variable effect of PRP in improving the pregnancy rate in women with RIF. Many of these reviews encompass both randomised controlled trials (RCTs) and observational studies, with each study involving a small number of participants. Due to the low quality of evidence, making clear recommendations becomes challenging. This umbrella review aimed to comprehensively synthesise and critically evaluate the available evidence from systematic reviews and meta-analyses on the use of PRP in women with RIF, assessing its effectiveness in improving reproductive outcomes and examining the quality and overlap of the existing evidence base. This article aims to guide clinicians in the use of PRP in women experiencing RIF.

## Methodology

We followed PRISMA guidelines (7) to report this review. This umbrella review included systematic reviews that had primarily incorporated RCTs. Systematic reviews were assessed for inclusion based on their “explicit, reproducible methodology,” comprehensive search methodology, acceptable methods for assessing the validity of included studies, evaluation of the risk of bias (ROB), and assessment of the certainty of the evidence. In addition, protocol registration status was considered as part of the inclusion criteria, with preference given to studies with prospectively registered protocols.

### Inclusion

Women of reproductive age diagnosed with RIF and planned for an *in vitro* fertilisation (IVF) cycle with fresh or frozen embryos were included. We exclusively focused on systematic reviews, with or without meta-analysis, that specifically addressed the role of PRP in the context of RIF and included RCTs. Eligible systematic reviews with clinical trials, comparing the use of PRP and routine care or no treatment in RIF patients undergoing IVF, with a clear definition of the diagnostic criteria for RIF, were included. Inclusion criteria also required a detailed description of the intervention, well-defined outcome(s), and the availability of full-text articles in the English language.

### Intervention

Study group: PRP in addition to the standard treatment for embryo transfer.

Control group: Placebo-controlled group; patients treated with other standard treatments such as gonadotropins, oestrogen, letrozole, colony-stimulating factor, granulocyte, sildenafil citrate, and tamoxifen; or a no-treatment group.

Primary outcome:

1. Live birth rate (LBR): Number of live births per cycle started.
2. Clinical pregnancy rate (CPR): Defined as the number of gestational sacs observed or foetal heartbeat identified.

Secondary outcome:

1. Chemical pregnancy rate: Defined as cases showing a positive pregnancy test.
2. Implantation rate: Defined as the number of gestational sacs observed divided by the number of embryos (cleavage-stage or blastocysts) transferred in one cycle.
3. Miscarriage rate: Defined as pregnancy leading to miscarriage in less than 12 weeks of gestation per total number of pregnancies.
4. Adverse drug events or reactions to PRP.

### Exclusion

We excluded all other types of studies (narrative reviews, systematic reviews of case reports/case series/short communications/animal studies, and observational studies only). Systematic reviews that did not describe (at a minimum) the search strategy, inclusion criteria, and quality assessment methods were excluded.

In addition, reviews involving studies conducted on women with known refractory endometrium (not responding to hormonal treatment) or tubercular pathology of the endometrium; uterine malformations such as septate/bicornuate uterus; fibroid or adenomyoma distorting the endometrial cavity; the presence of hydrosalpinx visible on ultrasound; or Asherman's syndrome were also excluded.

Search Strategy: A thorough web search using the keywords “PRP” OR “Platelet Rich Plasma” AND “RIF” OR recurrent Implantation failure OR unexplained RIF AND “Systematic review” OR “Meta-analysis” was performed by two independent reviewers (AC and SM). The search was conducted on Medline, PubMed, EMBASE, Web of Science, OVID, JBI Evidence Synthesis, the Cochrane Database of Systematic Reviews, DARE, and the PROSPERO register to identify systematic reviews addressing the research question from inception to date. The detailed search strategy is provided in Supplementary Table 1. Initially, title and abstract screening was carried out, followed by the second phase of full-text screening using the inclusion and exclusion criteria by two independent reviewers (AC and SM), and any controversy was resolved by a third reviewer (HK). Finally, a search of repositories of systematic reviews and the reference list of all included reviews, and a grey literature search was conducted to include any missing data.

Quality appraisal: The methodological quality of all included reviews was assessed independently by two reviewers using the AMSTAR tool, which consists of 11 items (8). A third reviewer was consulted in case of a difference of opinion. AMSTAR comprises 11 domains, seven of which are classified as critical due to their substantial impact on confidence in the conclusions drawn from systematic reviews. These critical domains encompass a range of crucial aspects, including the registration of the review protocol, the appropriateness of the search strategy, the reason for excluding specific studies, the risk-of-bias assessment of the included studies and its influence on the systematic review's conclusions, the method used for evidence synthesis, and the consideration of publication bias.

### Data extraction

Data for this umbrella review was extracted by compiling descriptive characteristics from the included systematic reviews, meta-analyses, or other studies. These descriptive variables included details such as the first author's name, publication year, study setting, the number of RCTs and observational studies included in the systematic review, characteristics of the study participants, details of interventions and comparators used, the outcomes assessed, the time of PRP administration in different studies, the route of PRP administration (endometrial/sub-endometrial), and the number of PRP doses administered.

### Data synthesis

Before compiling the results, a corrected covered area (CCA) metric was used to evaluate the degree of overlap among primary studies across the systematic reviews. This analysis is critical, as the presence of overlapping studies can introduce potential biases (9).

To calculate CCA, a citation matrix of primary studies was produced and included in our review. The CCA is a metric used in umbrella reviews to quantify the degree of overlap in primary studies across included systematic reviews. It is calculated using the formula:

$$\begin{array}{rl} & \mathrm{CCA}=N_{r}-N_{s}\\ & \;\;\;(R\times N_{s})-N_{s} \end{array}$$

where

- *N_r_* =total number of included references across all reviews
- *N_s_* =number of unique primary studies
- *R* = number of reviews

A CCA score of 0%–5% indicates slight overlap, 6%–10% moderate, 11%–15% high, and >15% very high overlap (9). A high CCA may signal redundancy and potential bias in umbrella reviews.

The compilation of evidence was presented in both narrative and tabular formats. We have provided a table that outlines the particulars of each systematic review included in our umbrella review. These particulars include information such as the target population, intervention studied, assessed outcomes, comparator, the number of primary studies and participants involved, the search databases used with their respective dates, and reported effect estimates, including risk ratios (RR), odds ratios (OR), hazard ratios (HR), mean difference (MD), standardised mean difference (SMD), or similar metrics, along with their corresponding confidence intervals (CIs). In addition, the table included details on heterogeneity, publication bias, final findings, quality assessments, and a summary of the risk of bias identified in the primary studies. For systematic reviews that involved both RCTs and observational studies, data were extracted only from the RCTs. We have utilised a narrative approach to succinctly summarise the evidence for each outcome, complemented by tabular formats wherever applicable for better understanding. When there was a higher level of overlap among reviews, preference was given to the results of the highest-rated systematic review according to AMSTAR guidelines. In cases where discrepancies arose between the outcomes of recent and high-quality systematic reviews, a re-analysis of the primary data was done to reach conclusive findings.

One of the included systematic reviews was co-authored by members of the present review team, and this has now been explicitly disclosed. To minimise the risk of bias, we implemented the following safeguards: (i) study selection was conducted independently by two reviewers, with any disagreements resolved by a third reviewer not involved in the overlapping review; (ii) risk of bias/quality assessment for the overlapping review was performed independently by reviewers with no involvement in that study; (iii) data extraction was carried out in duplicate by independent reviewers; and (iv) narrative synthesis and interpretation were undertaken with oversight from senior authors not associated with the overlapping publication.

If newer primary studies required data synthesis, a meta-analysis was conducted if more than three studies were available for each outcome. The meta-analysis involved pooling the effect sizes from each study using a random-effects model. Heterogeneity was assessed using the I2 statistic. Statistical analysis was performed by R software version 4.4.

The quality of evidence was reported using the Grading of Recommendations, Assessment, Development, and Evaluation (GRADE) criteria in the included systematic review. GRADE was reported as very low, low, moderate, and high.

Although the GRADE assessment is an important component of evidence synthesis, in the present umbrella review, *de novo* meta-analysis was based on aggregated data derived from previously published systematic reviews, rather than primary studies. Due to the lack of access to consistent, study-level data (e.g., detailed risk of bias domains, inconsistency, and imprecision across individual trials), a robust and reliable GRADE assessment for the pooled outcomes could not be performed.

## Results

**Figure 1:**
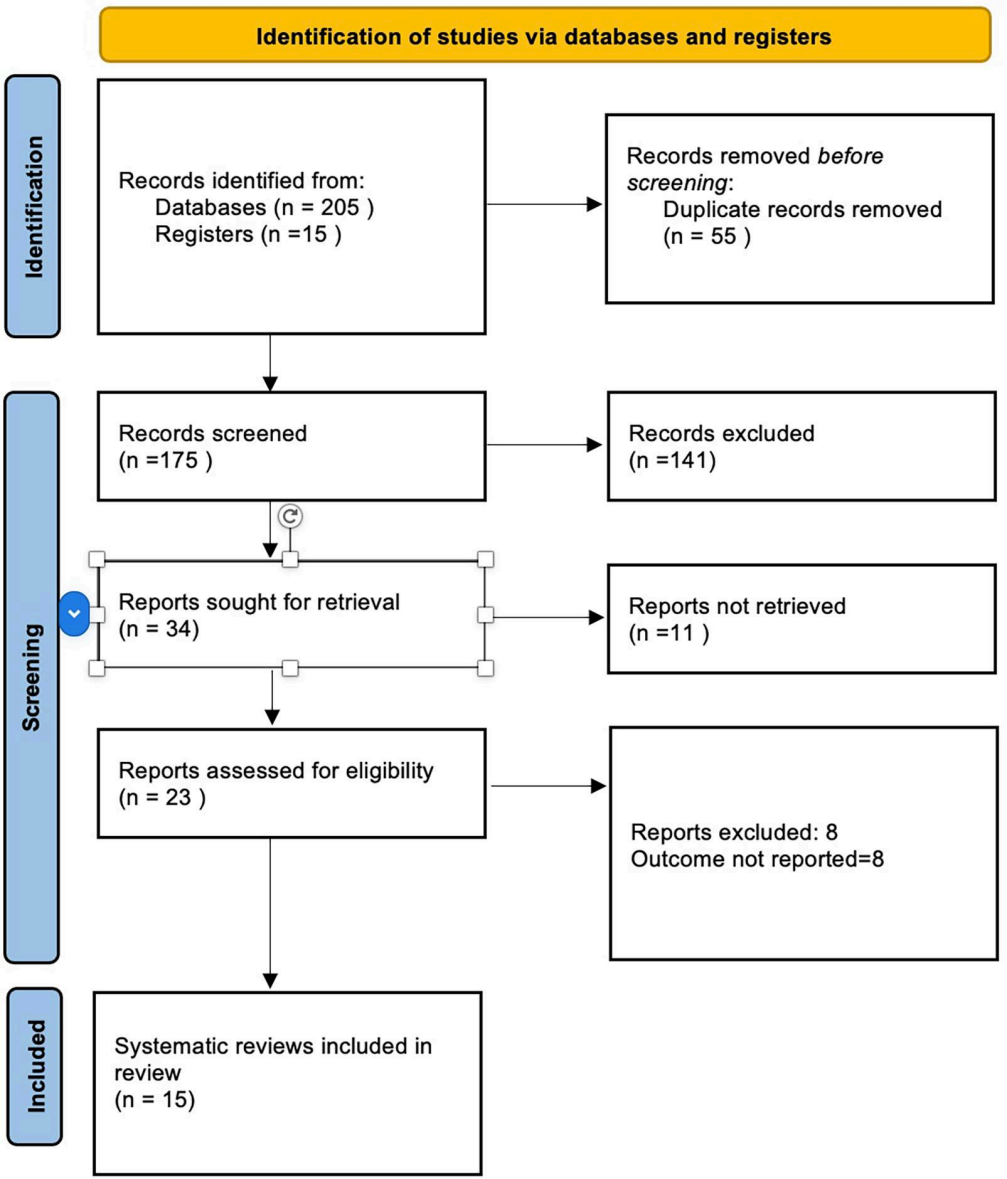
PRISMA flowchart of the screened studies.

The comprehensive literature search identified 230 records. After removing duplicates, 175 records were screened. Based on title/abstract screening, 141 records were excluded because they did not meet the inclusion criteria; these were either only RIF studies or PRP in IVF in general. Subsequently, 15 systematic reviews were assessed for eligibility and included in this umbrella review (4–6, 10–21). The details of the search results are illustrated in the PRISMA flow chart (Figure 1), along with the reasons for the exclusion of studies. All individual studies included in these systematic reviews were published between 2021 and 2024 (22–35).

The extracted data included study characteristics and outcome data for each study that met the inclusion criteria (Table 1).

**Table 1:** Details of the individual studies.

Study	Country	Year	Type of study	Number of studies included	Definition of RIF	PRP preparation protocol	Population	Intervention	Control	Outcomes assessed	Exclusion	Risk of bias	GRADE
Liu 2022	China	December 2022	RCTs and cohort	8 studies, 1219 participants	Implantation failure (variable definitions)	Autologous PRP; centrifugation methods differ	Infertile women who experienced one or more implantation failures	PRP administration before or after embryo transfer	No intervention or placebo	CPR- significant increase (OR 2.24, CI = 1.41–3.54, *p* = 0.0006, I = 61%), 2. LBR -5.76, CI = 1.55- 21.44, *p* = 0.009, I=85%), 3. miscarriage rate significantly less in those with PRP (OR = 0.18, CI = 0.05–0.63, *p* = 0.007, I=68%, no difference in multiple pregnancy rate)	Non-English language, details of research/results not available, review articles, editorials, case reports, and conference abstracts	High ROB for blinding and allocation concealment	NA
Li Muzi, 2022	China	September 2022	Cohort (4) and RCTs (6)	10 studies	Unexplained RIF (commonly ≥3 failed transfers with good embryos)	Autologous PRP; protocol varies across included trials	RIF, only English literature	PRP administration before or after embryo transfer	No intervention or granulocyte colony-stimulating factor (GCSF) infusion	CPR better in PRP group (*n* = 10, RR = 1.79, CI = 1.55 to 2.06, *p* < 0.01, I = 40%), LBR significantly better with PRP (*n* = 04, RR 2.92, CI = 2.22–3.85, *p* < 0.01, I = 83%), implantation rates significantly improved with PRP (*n* = 8, RR = 1.77, CI = 1.54–2.03, *p* < 0.01, I = 36%), no difference in miscarriage rates	Case reports, animal studies, cell experiments, bibliometric analysis, review, abstracts, self-pro-post studies, poor quality literature	High ROB for allocation concealment	NA
Eduardo Anitua et al		2023	RCTs	12 RCTs, 10 RCTs for meta-analysis	Embryo transfer failure	Standard PRP preparation (Anitua protocol referenced in some cases)	Women undergoing assisted reproduction with a history of embryo transfer failure	Intervention was intrauterine PRP infusion before embryo implantation.	No intervention or placebo	Repeated implantation failure: (A) clinical pregnancy rate 2.18 (1.76–2.70) 7 RCTs; (B) implantation rate, 1.57 (1.57–2.30) 3 RCTs; (C) biochemical pregnancy rate, 1.91 (1.54–2.37) 4 RCTs; (D) live birth rate, 3.36 (0.84–13.45) 3 RCTs; and (E) miscarriage rate 1.71 (0.54–5.48) 3 RCTs. Thin endometrium: (A) clinical pregnancy rate, 2.33 (0.98–5.44) 1 RCT (B)		Overall risk of bias was low in seven studies, moderate in one study, and high in two studies (a) Repeated implantation failure patients: not serious (b) Thin endometrium patients: serious	Clinical pregnancy rate = low, biochemical pregnancy rate = low, miscarriage rate = very low, live birth rate = very low
Busnelli		2021	Case–control, cohort, and RCTs	42 studies, 7 case–control studies, 12 prospective cohort studies, and 22 were RCTs	Mixed population (not limited to RIF)	Not specific to PRP alone (multiple interventions)	Women with failure to obtain a clinical pregnancy after at least three ET attempts	Different therapies and interventions including intrauterine autologous platelet-rich plasma (PRP) infusion		Primary outcomes were live birth rate (LBR) per patient and clinical pregnancy rate (CPR) per patient. “More intrauterine outcomes were implantation rate (IR) per embryo, multiple pregnancy rate (MPR) per patient, and miscarriage rate (MR) per patient. Two RCTs (155, 170) investigated whether the administration of intrauterine PRP could improve IVF outcomes in women with RIF. For primary outcomes, pooling of results showed a significantly increased chance of clinical pregnancy in treated women (fixed effects model, RR 2.45; 95% CI 1.55–3.86; *p* = 0.0001; I2 = 0%).		Mostly moderate, but some studies reported serious ROB.	Low
Deng	China	2022	Randomised controlled trials (RCTs) or cohort studies	Total 10: 8 English, 2 Chinese	RIF (definition varies across included studies)	Intrauterine PRP; preparation details variably reported	Infertile women who had experienced at least three implantation failures	Intrauterine perfusion of PRP before embryo transfer	Standard treatment or hormone replacement therapy (HRT)	Ten studies (six randomised controlled trials, four cohort studies) involving 1555 patients. Pregnancy outcomes improved in women treated with PRP compared with controls: clinical pregnancy rate (RR ¼ 1.96, 95% CI [1.67, 2.31], *p* < 0.00001, I2 ¼ 46%), chemical pregnancy rate (RR ¼ 1.79, 95% CI [1.54, 2.08], *p* < 0.00001, I2 ¼ 29%), implantation rate (RR ¼ 1.90, CI [1.50, 2.41], *p* < 0.00001, I2 ¼ 0%), live birth rate (RR ¼ 2.83, CI [1.45, 5.52], p ¼ 0.0007, I2 ¼ 83%), and abortion rate (RR ¼ 0.40, 95% CI [0.18, 0.90], p ¼ 0.03, I2 ¼ 59%)	(1) Animal or cell culture experiments; (2) single-arm studies or studies with a pre-post design; (3) case reports; or (4) conference abstracts	Seven were assessed as having a low risk of bias, and three were assessed as having a high risk of bias.	NA
Panda		2022	RCTs	4	RIF (typically ≥2 failed embryo transfers)	Autologous PRP; double centrifugation in most trials	Women with recurrent implantation failure	Intrauterine infusion of PRP		Implantation rate, clinical pregnancy rate. PRP significantly increased implantation and clinical pregnancy rates.		Low risk	Implantation rate = moderate, clinical pregnancy rate = moderate, chemical pregnancy rate = low
Maleki-Hagiagha		2023	RCTs and cohort	8	ART patients (not strictly RIF)	PRP preparation not standardised	Subfertile women undergoing ART	PRP infusion	Placebo, except one study that compared PRP versus granulocyte colony-stimulating factor			Moderate risk	NA
Solliman		2023	RCTs	8			38 women with more than 3 implantation failure attempts	Intrauterine infusion of autologous platelet-rich plasma (PRP)	Placebo or standard treatment	PRP significantly improved implantation, pregnancy, and birth outcomes in women with RIF. Chemical pregnancy, clinical pregnancy, and live birth rates were significantly higher in the PRP group. Miscarriage rates were lower, and multiple pregnancy rates were higher than those in controls.		Generally low; some studies had concerns about allocation concealment and performance bias.	NA
Huang	China	2023	RCTs, quasi-experimental, and cohort	14	RIF (commonly ≥2–3 failed cycles)	Intrauterine PRP infusion; limited protocol standardisation	Population of interest consisted of subfertile women undergoing assisted reproduction with any ovarian stimulation protocol, including no ovarian stimulation protocol	Intrauterine infusion of platelet-rich plasma (PRP) around the time of embryo transfer	Control group consisted of any other active intervention, no intervention, or a placebo.	No difference in miscarriage rates between women who received PRP and those who received placebo (*p* ≤ 0.90), chemical pregnancy (*p* ≤ 0.00), clinical pregnancy (*p* ≤ 0.001), and implantation rate (*p* ≤ 0.01)	Case–control studies, case series studies, cross-sectional studies, and animal or cell culture studies	For random allocation, all of the trials were found to have a minimal risk of bias. For one experiment, allocation concealment was shown to have a significant risk of bias. Half of the studies were found to have an uncertain risk of performance bias, while one research had a high risk of participant and personnel blinding. All except one of the studies were found to have a minimal probability of attrition bias.	NA
Kaur	India	2024	Randomised controlled trials		Unexplained RIF (clearly defined; e.g., ≥3 failed good-quality embryo transfers)	Autologous PRP; protocol described but varies across included RCTs.	Women of reproductive age diagnosed with RIF, i.e., who had at least two previous fresh or FET failures and were planned for an IVF cycle with fresh or frozen embryos.	Intrauterine instillation of PRP in addition to the standard treatment for embryo transfer.	Control group received a placebo; other routine treatments such as oestrogen, gonadotropins, letrozole, granulocyte colony-stimulating factor, sildenafil citrate, and tamoxifen; or no treatment.	Of these, 22 studies reached full-text screening and nine studies were included in the final review. We are uncertain about the effect of PRP due to the very low quality of evidence, and we have little confidence that the administration of PRP had any significant effect on improving the live birth rate in women with RIF (odds ratio [OR]: 7.32, 95% confidence interval [CI]: 4.54–11.81, I2 = 40%). Similarly, the quality of evidence was low for the CPR (OR: 3.20, 95% CI: 2.38–4.28, I2 = 0%). BPR- beneficial effect, (RR: 1.96, 95% CI: 1.63–2.36, I2 = 0%). MR- no significant effect (RR: 0.52, CI: 0.24–1.15, I2 = 61%). Endometrial thickness increased in women who received PRP vs women who received placebo after the intervention (*p* ≤ 0.001).	Studies such as observational studies, narrative reviews, case reports, case series and animal experiments, were excluded from the review. Clinical trials with multiple arms or with historical controls, quasi randomised studies were also excluded	80% of the studies have a low risk of bias for other biases, attrition bias, allocation concealment, detection bias, and reporting bias.	Biochemical pregnancy = very low, clinical pregnancy = low, live birth = very low, miscarriage = very low, ectopic pregnancy = very low, multiple pregnancy = very low
Shifu Hu et al, 2022	China	2022	RCTs	7	ART population (not exclusively RIF)	PRP from autologous blood; protocol inconsistent	Women with thin endometrium, implantation failure, or pregnancy failure undergoing treatment with assisted reproductive technology (ART).	Intrauterine infusion of autologous platelet-rich plasma (PRP).	No injection or placebo.	Of these, 22 studies reached full-text screening and nine studies were included in the final review. We are uncertain about the effect of PRP due to the very low quality of evidence and we have little confidence that the administration of PRP had any significant effect on improving the live birth rate in women with RIF (odds ratio [OR]: 7.32, 95% confidence interval [CI]: 4.54–11.81, I2 = 40%). Similarly, the quality of evidence was low for the CPR (risk ratio [RR]: 2.51; 95% CI: 2.0–3.13; *p* < 0.00001), chemical pregnancy (RR: 1.96; 95% CI: 1.58–2.45; *p* < 0.00001), LBR (RR: 7.03; 95% CI: 3.91–12.6; *p* < 0.00001), and IR (RR: 3.27; 95% CI: 1.42–7.52; *p* =0.005) were significantly higher in the women who received PRP infusion than in the control group. No significant differences were noted in the MR (RR: 0.98; 95% CI: 0.39–2.42; *p* = 0.96) between the two groups.	1. Review articles, commentaries, letters, or observational studies; 2. non-clinical trials; 3. not RCTs; and 4. reported inability to extract data from the literature	Risk of bias (ROB) results Random sequence generation: adequate in all studies Allocation concealment: Some studies lacked proper concealment. Blinding (participants and personnel): Not applied in all studies, increasing performance bias risk. Blinding (outcome assessment): Not consistently reported. Incomplete outcome data: No significant bias detected. Selective reporting: No major concerns. Overall ROB was moderate due to limitations in allocation concealment and blinding. Despite this, the studies were considered high quality for inclusion in the meta-analysis. Other biases: Some studies had geographical and author-related bias (multiple studies from the same research group). Conclusion on Risk of Bias Overall ROB was moderate due to limitations in allocation concealment and blinding. Despite this, the studies were considered high quality for inclusion in the meta-analysis.	NA
Ahmed M Maged et al, 2023	Egypt	2023	Randomised controlled trials (RCTs) mainly. A separate analysis for cohort or single-arm studies, whether prospective or retrospective, was done.	27 (13 RCTs and 14 non-RCTs)	Not explicitly uniform; generally ≥2–3 failed IVF cycles	Autologous PRP intrauterine infusion; centrifugation details not consistently reported	Women with previous implantation failure	PRP administration	No intervention/placebo	Implantation rate (IR): RCTs (6 studies, 886 participants): odds ratio (OR): 2.62 (95% CI: 1.83–3.76) Non-RCTs (3 studies, 587 participants): OR: 2.88 (95% CI: 1.14–7.25) Clinical pregnancy rate (CPR) RCTs (11 studies, 1,289 participants): OR: 2.46 (95% CI: 1.08–5.63) Non-RCTs (10 studies, 1,452 participants): OR: 2.39 (95% CI: 1.47–3.90) Chemical pregnancy rate RCTs (7 studies, 726 participants): OR: 2.92 (95% CI: 2.09–4.08) Non-RCTs (6 studies, 1,196 participants): OR: 1.49 (95% CI: 0.86–2.58) Ongoing pregnancy rate (OPR) RCTs (5 studies, 488 participants): OR: 2.78 (95% CI: 1.43–5.41) Non-RCTs (4 studies, 575 participants): OR: 4.09 (95% CI: 1.02–16.38) Live birth rate (LBR) RCTs (4 studies, 523 participants): OR: 4.35 (95% CI: 0.58–32.38) Non-RCTs (4 studies, 701 participants): OR: 4.18 (95% CI: 1.61–10.86) Endometrial thickness (ET) RCTs (4 studies, 307 participants): Mean difference (MD): 0.93 mm (95% CI: 0.59–1.27 mm) Non-RCTs (9 studies, 675 participants): MD: 1.16 mm (95% CI: 0.68–1.65 mm)	*In vitro* studies, animal studies, case reports, unclear methodologies	Mixed (low to high)	Implantation rate = moderate, clinical pregnancy rate = low, chemical pregnancy rate = high, ongoing pregnancy rate = low, live birth rate = very low, endometrial thickness = low
Shalma AM et al, 2023	Egypt	2023	14 RCTs, 3 non-RCTs, and 6 cohort studies	23	ART population ± RIF subgroup	PRP infusion; protocol heterogeneous	Subfertile women undergoing assisted reproduction (IVF/ICSI)	PRP administration	No PRP/placebo	1. Clinical pregnancy rate (CPR)- 23 studies, 2166 participants (1086 PRP cases, 1080 controls) Relative risk (RR): 1.84 (95% confidence interval (CI): 1.62–2.09); *p*-value: <0.00001 Conclusion: Women who received PRP had an 84% higher chance of achieving clinical pregnancy compared with those who did not receive PRP. 2. Live Birth Rate (LBR)- 10 studies, 1209 participants (617 PRP cases, 592 controls) RR: 1.75 (95% CI: 1.24–2.47); *p*-value: 0.001 Conclusion: PRP significantly increased the likelihood of a live birth by 75% compared with the control group. 3. Miscarriage rate—7 studies, 754 participants RR: 0.51 (95% CI: 0.36–0.72); *p*-value: 0.0002 Conclusion: PRP reduced the risk of miscarriage by 49%, suggesting a positive effect on pregnancy maintenance. 4. Implantation rate (IR)—8 studies, 1180 participants RR: 1.59 (95% CI: 1.36–1.86); *p*-value: <0.00001 Conclusion: PRP increased embryo implantation success by 59% compared with the control group. 5. Endometrial thickness (ET)- 12 studies, 1456 participants Mean difference (MD): 0.98 mm (95% CI: 0.65–1.31 mm); *p*-value: <0.00001 Conclusion: PRP administration resulted in a significant increase in endometrial thickness (∼1 mm), which is beneficial for implantation.	Abstracts, reviews, editorials, single-arm trials, case series, non-English studies	Mixed (low to high)	
Elgendy et al, 2023	Egypt	2023	1 Non RCT, 2 RCTs, 2 Retrospective cohort studies, 1 retrospective analysis, and 1 prospective cohort.	7	RIF (definition varies across studies)	PRP intrauterine infusion; heterogeneous methods	Infertile women with RIF	PRP administration		Clinical pregnancy rate, Biochemical pregnancy rate, and endometrial thickness 1. Clinical Pregnancy rate: 7 studies (3 RCTs), 59 in intervention and 25 in control group—RR = 1.79 CI 95% (1.37–2.32). 2. Biochemical pregnancy rate: 4 studies (71 in PRP group and 32 controls)—RR = 1.97 CI 95% (1.40–2.79). 3. Endometrial thickness—2 studies; RR = 1.79 CI 95% (1.13–2.44).	Age more than 40, endocrine and thyroid disorders, tubal infertility in HSG, cardiovascular, renal, or hepatic disorder, congenital uterine deformities, endometritis, tubal factors like hydrosalpinx	Low risk of bias	Not specified
Tingting Ma 2024		2024	9 RCTs PRP vs placebo, 2 RCTs PRP vs GCSF	11	RIF	PRP intrauterine infusion	Infertile women	PRP	Placebo/GCSF	CPR- (*n* = 23; 2166 participants (1086 PRP cases, 1080 controls); RR: 1.84 (CI: 1.62 – 2.09); *p* = <0.00001). LBR- (*n* = 10; 1209 participants (617 PRP cases, 592 controls); RR: 1.75 (CI: 1.24 – 2.47); *p* = 0.001). MR- (*n* = 7; 754 participants; RR: 0.51 (CI: 0.36 – 0.72); *p* = 0.0002); PRP reduced the risk of miscarriage by 49%. IR- (*n* = 8; 1180 participants; RR: 1.59 (95% CI: 1.36 – 1.86); *p* = <0.00001). ET- (*n* =12; 1456 participants). Mean Difference (MD): 0.98 mm (95% CI: 0.65 – 1.31 mm); *p*-value: <0.00001; PRP resulted in a significant increase in endometrial thickness (∼1 mm), beneficial for implantation.	Cluster RCTs and pseudo-randomised trials. For cross-over trials, we planned to include only data from the first phase (before cross-over).		
Liu K, 2022	China	December 2022	RCTs and cohort	08 studies, 1219 participants	Implantation failure (variable definitions)	Autologous PRP; centrifugation methods differ	Infertile women who experienced one or more implantation failures	PRP administration before or after embryo transfer	No intervention or placebo	CPR- significant increase in CPR (OR 2.24, CI = 1.41–3.54, *p* = 0.0006, I = 61%), 2. LBR -5.76, CI = 1.55- 21.44, *p* = 0.009, I=85%), 3. miscarriage rate significantly less in those with PRP (OR = 0.18, CI = 0.05–0.63, *p* = 0.007, I=68%, no difference in multiple pregnancy rate)	Non-English language, details of research/results not available, review articles, editorials, case reports, and conference abstracts	High ROB for blinding and allocation concealment	NA
Li Muzi, 2022	China	September 2022	Cohort (4) and RCTs (6)	10 studies	Unexplained RIF (commonly ≥3 failed transfers with good embryos)	Autologous PRP; protocol varies across included trials	RIF, only English literature	PRP administration before or after embryo transfer	No intervention or GCSF infusion	CPR better in PRP group (*n* = 10, RR = 1.79, CI = 1.55 to 2.06, *p* < 0.01, I = 40%), LBR significantly better with PRP (*n* = 04, RR 2.92, CI = 2.22–3.85, *p* < 0.01, I = 83%), implantation rates significantly improved with PRP (*n* = 8, RR = 1.77, CI = 1.54–2.03, *p* < 0.01, I = 36%), no difference in miscarriage rates	Case reports, animal studies, cell experiments, bibliometric analysis, review, abstracts, self-pro-post studies, poor quality literature	High ROB for allocation concealment	NA
Eduardo Anitua et al		2023	RCTs	12 RCTs; 10 RCTs for meta-analysis	Embryo transfer failure	Standard PRP preparation (Anitua protocol referenced in some cases)	Women undergoing assisted reproduction with a history of embryo transfer failure	Intrauterine PRP infusion before embryo implantation	Comparison (C) was to no intervention or placebo	Repeated implantation failure: (A) clinical pregnancy rate 2.18 (1.76–2.70) 7 RCTs (B) Implantation rate, 1.57 (1.57–2.30) 3 RCTs (C) biochemical pregnancy rate, 1.91 (1.54–2.37) 4 RCTs (D) live birth rate, 3.36 (0.84–13.45) 3 RCTs and (E) miscarriage rate 1.71 (0.54–5.48) 3 RCTs. Thin endometrium: (A) clinical pregnancy rate, 2.33 (0.98–5.44) 1 RCT (B)		Overall risk of bias was low in seven studies, moderate in one study, and high in two studies: a) repeated implantation failure patients: not serious; b) thin endometrium patients: serious	Clinical pregnancy rate = low, biochemical pregnancy rate = low, miscarriage rate = very low, live birth rate = very low
Busnelli		2021	Case–control, cohort, and RCTs	42 studies, 7 case–control studies, 12 prospective cohort studies and 22 were RCTs	Mixed population (not limited to RIF)	Not specific to PRP alone (multiple interventions)	Women with failure to obtain a clinical pregnancy after at least three ET attempts	Different therapies and interventions INCLUDING Intrauterine autologous platelet-rich plasma (PRP) infusion		Primary outcomes were live birth rate (LBR) per patient and clinical pregnancy rate (CPR) per patient. “More intrauterine outcomes were implantation rate (IR) per embryo, multiple pregnancy rate (MPR) per patient, and miscarriage rate (MR) per patient Two RCTs (155, 170) investigated whether administration of intrauterine PRP could improve IVF outcomes in women with RIF. Primary outcomes: pooling of results showed a significantly increased chance of clinical pregnancy in treated women (fixed effects model, RR 2.45; 95% CI 1.55–3.86; *p* = 0.0001; I2 = 0%)		Mostly moderate, but some studies reported serious ROB	Low
Deng	China	2022	Randomised controlled trials (RCTs) or cohort studies;	Total 10, 8 English, 2 Chinese	RIF (definition varies across included studies)	Intrauterine PRP; preparation details variably reported	Infertile women who had experienced at least three implantation failures	Intrauterine perfusion of PRP before embryo transfer	Standard treatment or HRT	Ten studies (six randomised controlled trials, four cohort studies) involving 1,555 patients were included. Pregnancy outcomes improved in women treated with PRP compared with controls: clinical pregnancy rate (RR ¼ 1.96, 95% CI [1.67, 2.31], *p* < 0.00001, I2 ¼ 46%), chemical pregnancy rate (RR ¼ 1.79, 95% CI [1.54, 2.08], *p* < 0.00001, I2 ¼ 29%), implantation rate (RR ¼ 1.90, CI [1.50, 2.41], *p* < 0.00001, I2 ¼ 0%), live birth rate (RR ¼ 2.83, CI [1.45, 5.52], p ¼ 0.0007, I2 ¼ 83%), and abortion rate (RR ¼ 0.40, 95% CI [0.18, 0.90], p ¼ 0.03, I2 ¼ 59%).	(1) Animal or cell culture experiments; (2) single-arm studies or studies with a pre-post design; (3) case reports; or (4) conference abstracts.	Seven studies were assessed as having a low risk of bias, and three were assessed as having a high risk of bias.	NA
Panda		2022	RCTs	4	RIF (typically ≥2 failed embryo transfers)	Autologous PRP; double centrifugation in most trials	Women with recurrent implantation failure	Intrauterine infusion of PRP		Implantation rate, clinical pregnancy rate PRP significantly increased implantation and clinical pregnancy rates.		Low risk	Implantation rate = moderate, clinical pregnancy rate = moderate, chemical pregnancy rate = low
Maleki-Hagiagha		2023	RCTs and cohort	8	ART patients (not strictly RIF)	PRP preparation not standardised	Subfertile women undergoing ART	PRP infusion	Placebo, except one study that compared PRP versus granulocyte colony-stimulating factor.			Moderate risk	NA
Solliman		2023	RCTs	8			38 women with more than 3 implantation failure attempts	Intrauterine infusion of autologous platelet-rich plasma (PRP)	Placebo or standard treatment	PRP significantly improved implantation, pregnancy, and birth outcomes in women with RIF. Chemical pregnancy, clinical pregnancy, and live birth rates were significantly higher in the PRP group. Miscarriage rates were lower, and multiple pregnancy rates were higher than those in controls.		Generally low; some studies had concerns about allocation concealment and performance bias.	NA
Huang	China	2023	RCTs, quasi-experimental, and cohort	14	RIF (commonly ≥2–3 failed cycles)	Intrauterine PRP infusion; limited protocol standardisation	Population of interest consisted of subfertile women undergoing assisted reproduction with any ovarian stimulation protocol, including no ovarian stimulation protocol.	Intrauterine infusion of platelet-rich plasma (PRP) around the time of embryo transfer	Control group consisted of any other active intervention, no intervention, or a placebo	No difference in miscarriage rates between women who received PRP and those who received placebo (*p* ≤ 0.90). Chemical pregnancy (*p* ≤ 0.00), clinical pregnancy (*p* ≤ 0.001), and implantation rate (*p* ≤ 0.01)	Case–control studies, case series studies, cross-sectional studies, and animal or cell culture studies.	For random allocation, all of the trials were	NA
Kaur	India	2024	Randomised controlled trials		Unexplained RIF (clearly defined; e.g., ≥3 failed good-quality embryo transfers)	Autologous PRP; protocol described but varies across included RCTs.	Women of reproductive age diagnosed with RIF, i.e., who had at least two previous fresh or FET failures and were planned for an IVF cycle with fresh or frozen embryos.	Intrauterine instillation of PRP in addition to the standard treatment for embryo transfer.	Control group received a placebo; other routine treatments such as oestrogen, gonadotropins, letrozole, granulocyte colony-stimulating factor, sildenafil citrate, and tamoxifen; or no treatment.	Of these, 22 studies reached full-text screening and nine studies were included in the final review. We are uncertain about the effect of PRP due to the very low quality of evidence, and we have little confidence that the administration of PRP had any significant effect on improving the live birth rate in women with RIF (odds ratio [OR]: 7.32, 95% confidence interval [CI]: 4.54–11.81, I2 = 40%). Similarly, the quality of evidence was low for the CPR (OR: 3.20, 95% CI: 2.38–4.28, I2 = 0%). BPR- beneficial effect, (RR: 1.96, 95% CI: 1.63–2.36, I2 = 0%). MR- no significant effect (RR: 0.52, CI: 0.24–1.15, I2 = 61%). Endometrial thickness increased in women who received PRP vs women who received placebo after the intervention (*p* ≤ 0.001)		80% of the studies have a low risk of bias for other biases, attrition bias, allocation concealment, detection bias, and reporting bias experiment, and allocation concealment	Biochemical pregnancy = very low, clinical pregnancy = low, live birth = very low, miscarriage = very low, ectopic pregnancy = very low, multiple pregnancy = very low
Shifu Hu et al, 2022	China	2022	RCTs	7	ART population (not exclusively RIF)	PRP from autologous blood; protocol inconsistent	Women with thin endometrium, implantation failure, or pregnancy failure undergoing treatment with assisted reproductive technology (ART).	Intrauterine infusion of autologous platelet-rich plasma (PRP).	No injection or placebo.	Of these, 22 studies reached full-text screening and nine studies were included in the final review. We are uncertain about the effect of PRP due to the very low quality of evidence, and we have little confidence that the administration of PRP had any significant effect on improving the live birth rate in women with RIF (odds ratio [OR]: 7.32, 95% confidence interval [CI]: 4.54–11.81, I2 = 40%). Similarly, the quality of evidence was low for the CPR (OR: 3.20, 95% CI: 2.38–4.28, I2 = 0%). BPR- beneficial effect, (RR: 1.96, 95% CI: 1.63–2.36, I2 = 0%). MR- no significant effect (RR: 0.52, CI: 0.24–1.15, I2 = 61%). Endometrial thickness increased in women who received PRP vs women who received placebo after the intervention (*p* ≤ 0.001)	1. Review articles, commentaries, letters, or observational studies; 2. non-clinical trials; 3. not RCTs; and 4. reported inability to extract data from the literature.	Risk of bias (ROB) results Random sequence generation: Adequate in all studies. Allocation concealment: Some studies lacked proper concealment. Blinding (participants and personnel): Not applied in all studies, increasing performance bias risk. Blinding (outcome assessment): Not consistently reported. Incomplete outcome data: No significant bias detected. Selective reporting: No major concerns. Overall ROB was moderate due to limitations in allocation concealment and blinding. Despite this, the studies were considered high quality for inclusion in the meta-analysis. Other biases: Some studies had geographical and author-related bias (multiple studies from the same research group). Conclusion on risk of bias: Overall ROB was moderate due to limitations in allocation concealment and blinding. Despite this, the studies were considered high quality for inclusion in the meta-analysis.	NA
Ahmed M Maged et al, 2023	Egypt	2023	Randomised controlled trials (RCTs) mainly. A separate analysis for cohort or single-arm studies, whether prospective or retrospective, was done.	27 (13 RCTs and 14 Non-RCTs)	Not explicitly uniform; generally ≥2–3 failed IVF cycles	Autologous PRP intrauterine infusion; centrifugation details not consistently reported	Women with previous implantation failure	PRP administration	No intervention/placebo	1. Implantation rate (IR) RCTs (6 studies, 886 participants): odds ratio (OR): 2.62 (95% CI: 1.83–3.76) Non-RCTs (3 studies, 587 participants): OR: 2.88 (95% CI: 1.14–7.25) 2. Clinical pregnancy rate (CPR) RCTs (11 studies, 1,289 participants): OR: 2.46 (95% CI: 1.08–5.63) Non-RCTs (10 studies, 1,452 participants): OR: 2.39 (95% CI: 1.47–3.90) 3. Chemical pregnancy rate RCTs (7 studies, 726 participants): OR: 2.92 (95% CI: 2.09–4.08) Non-RCTs (6 studies, 1,196 participants): OR: 1.49 (95% CI: 0.86–2.58) 4. Ongoing pregnancy rate (OPR) RCTs (5 studies, 488 participants): OR: 2.78 (95% CI: 1.43–5.41) Non-RCTs (4 studies, 575 participants): OR: 4.09 (95% CI: 1.02–16.38) 5. Live birth rate (LBR) RCTs (4 studies, 523 participants): OR: 4.35 (95% CI: 0.58–32.38) Non-RCTs (4 studies, 701 participants): OR: 4.18 (95% CI: 1.61–10.86) 6. Endometrial thickness (ET) RCTs (4 studies, 307 participants): mean difference (MD): 0.93 mm (95% CI: 0.59–1.27 mm) Non-RCTs (9 studies, 675 participants): MD: 1.16 mm (95% CI: 0.68–1.65 mm)	*In vitro* studies, animal studies, case reports, unclear methodologies	Mixed (low to high)	Implantation rate = moderate, clinical pregnancy rate = low, chemical pregnancy rate = high, ongoing pregnancy rate = low, live birth rate = very low, endometrial thickness = low
Shalma AM et al, 2023	Egypt	2023	14 RCTs, 3 non-RCTs, 6 cohort studies	23	ART population ± RIF subgroup	PRP infusion; protocol heterogeneous	Subfertile women undergoing assisted reproduction (IVF/ICSI)	PRP administration	No PRP/placebo	Clinical pregnancy rate (CPR)-23 studies, 2166 participants (1,086 PRP cases, 1,080 controls) Relative risk (RR): 1.84 (95% Confidence interval (CI): 1.62–2.09); *p*-value: <0.00001 Conclusion: Women who received PRP had an 84% higher chance of achieving clinical pregnancy compared with those who did not receive PRP. 2. Live birth rate (LBR)- 10 studies, 1209 participants (617 PRP cases, 592 controls) RR: 1.75 (95% CI: 1.24–2.47); *p*-value: 0.001 Conclusion: PRP significantly increased the likelihood of a live birth by 75% compared with the control group. 3. Miscarriage rate—7 studies, 754 participants RR: 0.51 (95% CI: 0.36–0.72); *p*-value: 0.0002 Conclusion: PRP reduced the risk of miscarriage by 49%, suggesting a positive effect on pregnancy maintenance. 4. Implantation rate (IR)—8 studies, 1180 participants RR: 1.59 (95% CI: 1.36–1.86); *p*-value: <0.00001 Conclusion: PRP increased embryo implantation success by 59% compared with the control group. 5. Endometrial thickness (ET)- 12 studies, 1456 participants Mean difference (MD): 0.98 mm (95% CI: 0.65–1.31 mm); *p*-value: <0.00001 Conclusion: PRP administration resulted in a significant increase in endometrial thickness (∼1 mm), which is beneficial for implantation.	Abstracts, reviews, editorials, single-arm trials, case series, non-English studies	Mixed (low to high)	
Elgendy et al, 2023	Egypt	2023	1 non-RCT, 2 RCTs, 2 Retrospective cohort studies, 1 retrospective analysis and 1 prospective cohort.	7	RIF (definition varies across studies)	PRP intrauterine infusion; heterogeneous methods	Infertile women with RIF	PRP administration		Clinical pregnancy rate, biochemical pregnancy rate and endometrial thickness 1. Clinical pregnancy rate: 7 studies (3 RCTs), 59 in intervention and 25 in control group—RR = 1.79 CI 95% (1.37–2.32). 2. Biochemical pregnancy rate: 4 studies (71 in PRP group and 32 controls)—RR = 1.97 CI 95% (1.40-2.79). 3. Endometrial thickness—2 studies; RR = 1.79 CI 95% (1.13–2.44).	Age more than 40, endocrine and thyroid disorders, tubal infertility (HSG), cardiovascular, renal, or hepatic disorder, congenital uterine deformities, endometritis, tubal factors like hydrosalpinx	Low risk of bias	Not specified
Tingting Ma 2024	2024	9 RCTs PRP vs placebo, 2 RCTs PRP vs GCSF	11	RIF	PRP intrauterine infusion	Infertile women	PRP	Placebo/GCSF	Clinical pregnancy rate (CPR) RR = 1.78 (95% CI: 1.51–2.11), *p* < 0.00001 Significant improvement with PRP Live birth rate (LBR) RR = 2.62 (95% CI: 0.87–7.92), *p* = 0.09 Not statistically significant Implantation rate (IR) RR = 1.79 (95% CI: 1.39–2.29), *p* < 0.00001 Significant improvement Spontaneous abortion rate (miscarriage) RR = 0.51 (95% CI: 0.30–0.81), *p* = 0.01 Significant reduction Endometrial thickness (ET) SMD = 0.39 (95% CI: −0.23 to 1.01), *p* = 0.22 Not statistically significant	Cluster RCTs and pseudo-randomised controlled trials. For cross-over trials, we planned to include only data from the first phase (before cross-over). CPR- (*n* = 23; 2166 participants (1086 PRP cases, 1080 controls); RR: 1.84 (CI: 1.62 – 2.09); *p* = <0.00001). LBR- (*n* = 10; 1209 participants (617 PRP cases, 592 controls); RR: 1.75 (CI: 1.24 – 2.47); *p* = 0.001). MR- (*n* = 7; 754 participants; RR: 0.51 (CI: 0.36 – 0.72); *p* = 0.0002); PRP reduced the risk of miscarriage by 49%. IR- (*n* = 8; 1180 participants; RR: 1.59 (95% CI: 1.36 – 1.86); *p* = <0.00001). ET- (*n* =12; 1456 participants). Mean Difference (MD): 0.98 mm (95% CI: 0.65 – 1.31 mm); *p*-value: <0.00001; PRP resulted in a significant increase in endometrial thickness (∼1 mm), beneficial for implantation.			NA

CPR, clinical pregnancy rate; LBR, live birth rate; BPR, biochemical pregnancy rate; IR, implantation rate; MR, miscarriage rate; ET, endometrial thickness; OPR, ongoing pregnancy rate; OR, odds ratio; RR, relative Relative Risk.

All included systematic reviews were individually assessed for duplicated trials in their analysis using the CCA index. A colour-coded table was prepared for all trials in the included systematic reviews, with green indicating duplicate trials (Table 2). The calculated CCA index for the included systematic reviews was 42%. Due to the high CCA index, the effect estimates of the systematic reviews were not pooled or meta-analysed. However, quantitative analysis was conducted for all outcomes by extracting data from each trial within all included systematic reviews to overcome the problem of overestimating effect size from similar trials being included in systematic reviews. The results of the outcomes reported by these systematic reviews are descriptively narrated in Table 1.

**Table 2:** Corrected covered area (CCA) to assess the degree of overlap among primary studies of included reviews.

Primary studies of systematic reviews	Included systematic reviews														
	Anitua et al. (19)	Busnelli et al. (10)	Deng et al. (11)	Huang et al. (13)	Kaur et al. (6)	Hu et al. (12)	Liu et al. (14)	Ma and Pu (15)	Li et al. (5)	Solliman	Panda et al. (16)	Shamla et al. (17)	Elsalam Elgendy et al. (21)	Maleki-Hagiagha et al. (20)	Maged et al. (4)
Allahveisi et al., 2020															
Bakhsh et al., 2021															
Ershadi et al., 2022															
Nazari et al., 2020															
Nazari et al., 2021															
Nazari et al., 2022															
Zargar et al., 2021															
Rageh, K. A. I. 2020															
Safdarian, L. 2022															
Zamaniyan, M. 2020															
Obidniak 2017															
Dawood 2022															
Baybordi et al. 2022															
Elsarman 2022															

### Narrative results of included systematic reviews

A total of 15 systematic reviews (4, 5, 6, 10–21) and meta-analyses conducted between 2021 and 2024 consistently reported improved reproductive outcomes with intrauterine PRP administration in women undergoing ARTs, particularly those with RIF (Table 1):

1. LBR: Despite variations in the strength of evidence, most reviews showed a significantly higher LBR in the PRP group. For instance, Maged et al. (4) and Shalma et al. (17) reported increased LBRs with RRs up to 4.35 (RCTs) and 4.18 (non-RCTs), although Kaur et al. (4) and Hu et al. (12) noted that the certainty of evidence was low to very low because of study heterogeneity and risk of bias.
2. CPR: Most reviews demonstrated a statistically significant increase in CPR among women receiving PRP compared with controls. RR estimates ranged from 1.79 to 2.46, with studies by Liu et al. (14), Anitua et al. (19), Maged et al. (4), and Shalma et al. (17) reporting ORs or RRs above 2.0, indicating that PRP nearly doubled the likelihood of clinical pregnancy. Ma and Pu (15) confirmed this trend, with an RR of 1.84 (95% CI: 1.62–2.09). However, we cannot comment on the strength of evidence as the GRADE was reported in only five systematic reviews (4, 6, 10, 16, 19).
3. Implantation rate (IR): Several systematic reviews, including Deng et al. (11), Anitua et al. (19), and Maged et al. (4), demonstrated improved implantation rates with PRP, with reported RRs ranging from 1.57 to 2.88. In particular, Shalma et al. (17) showed a 59% increase in IR, and Huang et al. (13) corroborated this with significant *p*-values (≤0.01). However, it is difficult to comment on the strength of evidence as the GRADE was reported in only five systematic reviews (4, 6, 10, 16, 19).
4. Biochemical and ongoing pregnancy rates: Some studies, including Anitua et al. (19) and Maged et al. (4), included biochemical and ongoing pregnancy rates (4, 19). These outcomes also favoured PRP administration, with biochemical pregnancy ORs ranging from 1.91 to 2.92 and ongoing pregnancy ORs from 2.78 to 4.09, although the certainty of evidence varied.
5. Miscarriage rate: A reduction in the miscarriage rate was reported across several reviews. Liu et al. (14) found significantly fewer miscarriages in the PRP group (OR = 0.18, 95% CI: 0.05–0.63), while Shalma et al. (17) reported a 49% decrease (RR = 0.51). Ma and Pu (15) supported these findings, indicating a consistent trend towards reduced miscarriage with PRP. However, due to missing GRADE reporting by the majority of reviews (17, 21, 36), it is not possible to comment on the strength of evidence.
6. Endometrial thickness (ET): Improvement in endometrial thickness post-PRP infusion was a consistent finding in reviews such as those by Maged et al. and Shalma et al. (4, 17). The mean difference ranged from 0.93 to 1.16 mm, indicating a moderate but statistically significant enhancement in endometrial receptivity (*p* < 0.00001). Some reviews included studies with unexplained RIF, i.e., excluding cases with thin endometrium (Kaur et al), while others included studies with thin endometrium as well (12, 19).

Overall, the narrative synthesis across these systematic reviews supports the potential benefit of intrauterine PRP in improving implantation and pregnancy outcomes in women with RIF, although the certainty of evidence is either low or not reported in the various systematic reviews. While clinical pregnancy and implantation rates consistently improved, heterogeneity in live birth outcome definitions and moderate to low GRADE quality across reviews suggest that further high-quality, well-controlled trials are warranted to confirm the long-term reproductive efficacy of PRP. Nonetheless, miscarriage rates decreased and endometrial thickness improved, reinforcing PRP's favourable role in endometrial receptivity.

### Meta-analysis of the primary studies in the included systematic reviews (after excluding overlaps)

A quantitative analysis of data extracted from a total of 12 studies (reported in included systematic reviews) was compiled in the meta-analysis (Supplementary Table 2).

### Live birth rate

**Figure 2:**
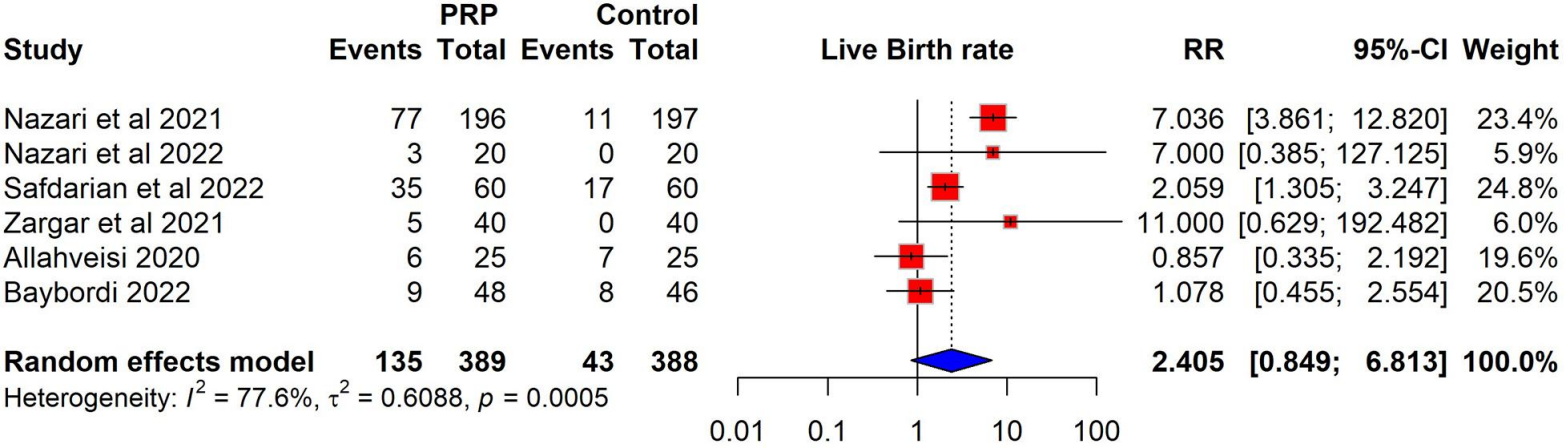
Pooled live birth rate following intrauterine platelet-rich plasma (PRP) infusion in women with unexplained recurrent implantation failure (RIF). Forest plot displaying pooled estimates of live birth rate from systematic reviews and meta-analyses evaluating the efficacy of PRP in women with unexplained RIF. Each box represents the effect size from an individual meta-analysis, with horizontal lines indicating the 95% confidence intervals (CIs). The size of the box reflects the weight of the study. The diamond represents the overall pooled estimate with its 95% CI. Statistical heterogeneity was assessed using the I^2^ statistic.

Six studies reported LBR as an outcome, but the definition of live birth varied across studies. Gestation beyond 24 weeks of pregnancy was considered an LBR in Nazari et al., whereas Nazari et al., Safdarian et al., and Zargar et al. (23, 27, 28, 35) defined LBR as delivery of a live baby after viability. We are uncertain about the effect of PRP because of the very low quality of evidence, and we have little confidence that the administration of PRP had any significant effect on improving LBR in women with RIF undergoing IVF treatment (RR: 2.40, 95% CI: 0.849–6.813, *I*^2^ = 77.6%) (Figure 2).

### Clinical pregnancy rate

**Figure 3:**
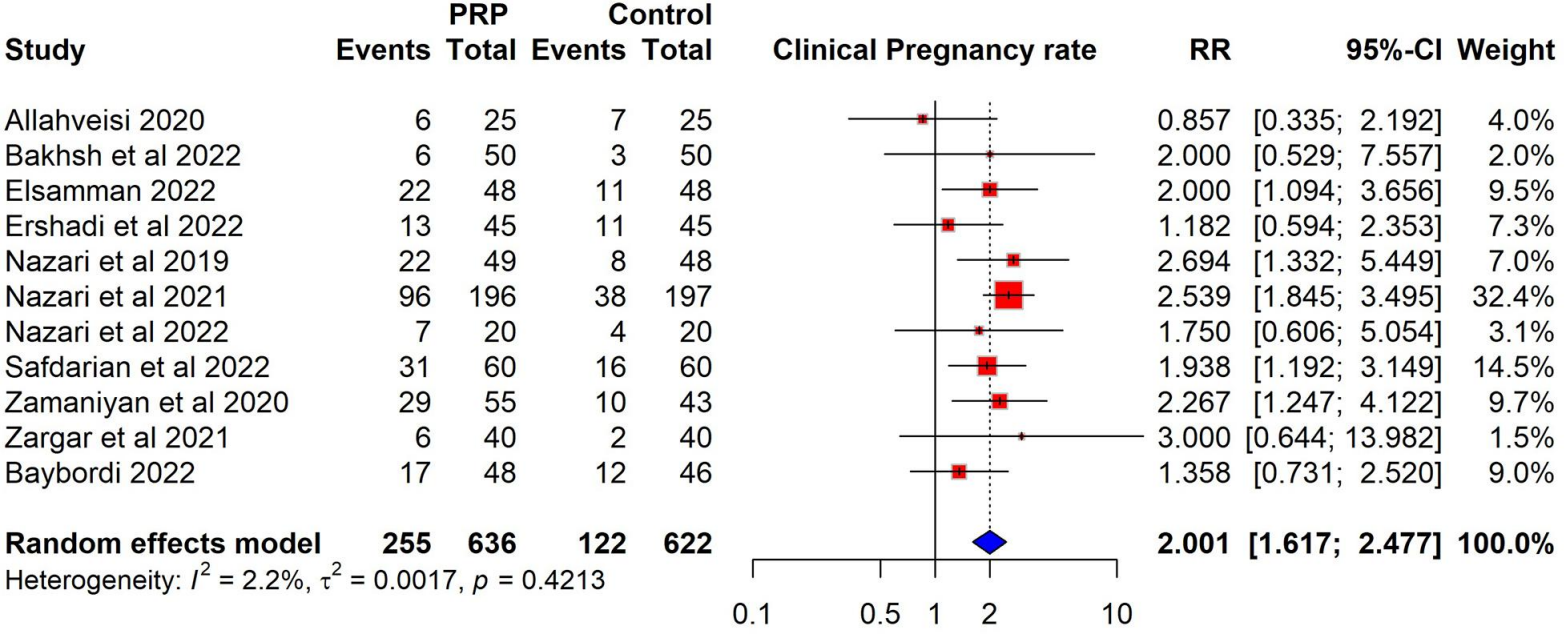
Pooled clinical pregnancy rate following intrauterine platelet-rich plasma (PRP) infusion women with unexplained recurrent implantation failure (RIF). Forest plot displaying pooled estimates of live birth rate from systematic reviews and meta-analyses evaluating the efficacy of PRP in women with unexplained RIF. Each box represents the effect size from an individual meta-analysis, with horizontal lines indicating the 95% confidence intervals (CIs). The size of the box reflects the weight of the study. The diamond represents the overall pooled estimate with its 95% CI. Statistical heterogeneity was assessed using the I^2^ statistic.

A total of 11 RCTs reported clinical pregnancy rates. Of the 11 studies included, three did not report the statistical values for the outcomes and thus could not be pooled for quantitative synthesis. Therefore, only eight studies were used for quantitative synthesis. The pooled effect of the eight studies showed some benefit of PRP on clinical pregnancy rate, but the quality of evidence was very low and the true effect could be substantially different from the effect estimate that administration of PRP had any significant effect on clinical pregnancy rate (RR: 2.00, 95% CI: 1.617–2.47, *I*^2^ = 2.2%) (Figure 3).

**Figure 4:**
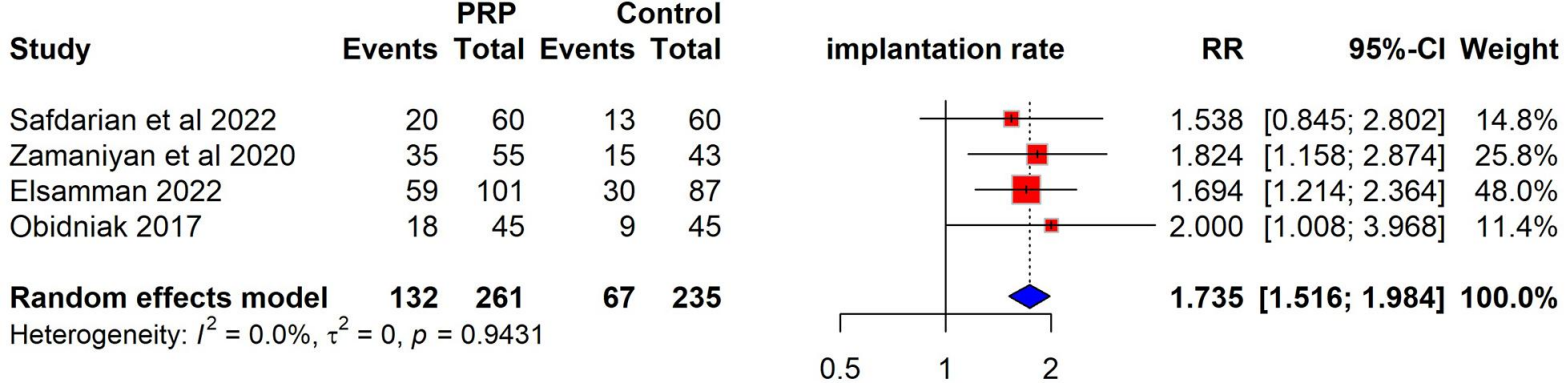
Pooled implantation rate following intrauterine platelet-rich plasma (PRP) infusion women with unexplained recurrent implantation failure (RIF).

Implantation rate: Only four clinical trials reported implantation rates. The pooled effect of four trials showed a significant beneficial effect of PRP on implantation rate (RR: 1.73, 95% CI: 1.516–1.984, *I*^2^ = 0.0%) (Figure 4).

### Biochemical pregnancy rate

**Figure 5:**
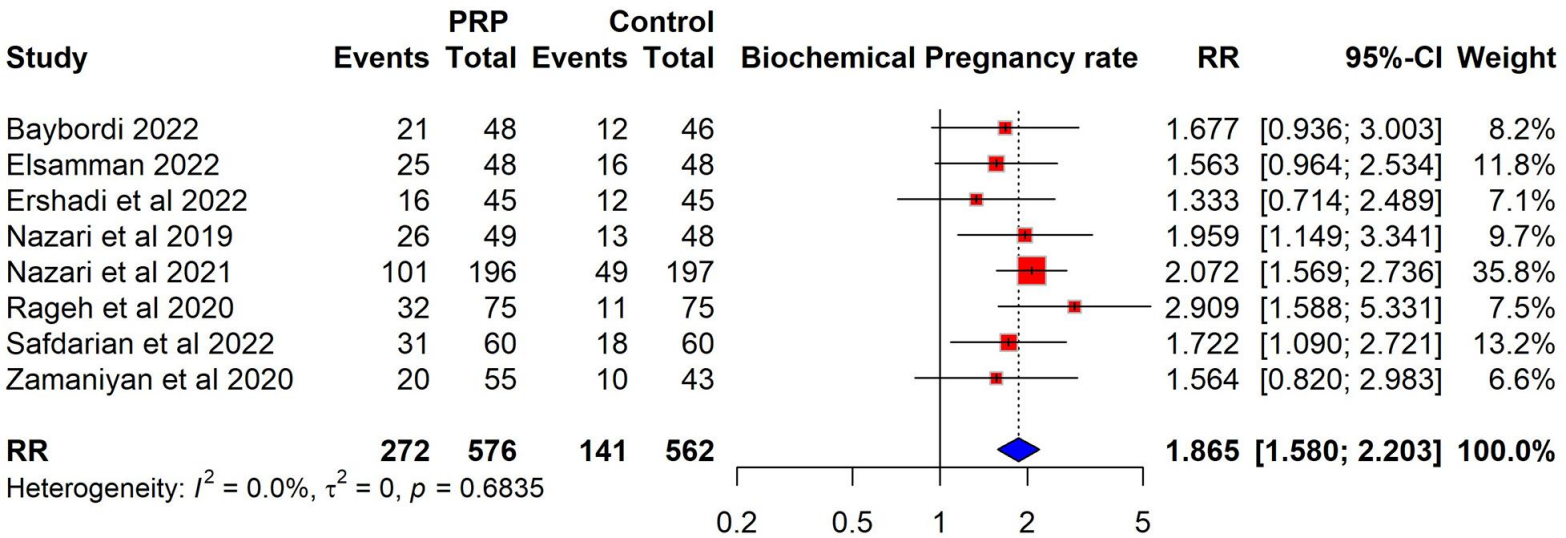
Pooled biochemical pregnancy rate following intrauterine platelet-rich plasma (PRP) infusion women with unexplained recurrent implantation failure (RIF).

Eight RCTs reported a biochemical pregnancy rate. The pooled effect of these eight trials showed a significant beneficial effect of PRP on biochemical pregnancy rate, but the quality of evidence was very low, and our confidence in the effect estimate is limited (RR: 1.86, 95% CI: 1.58–2.20, *I*^2^ = 0%) (Figure 5).

### Miscarriage rate

**Figure 6:**
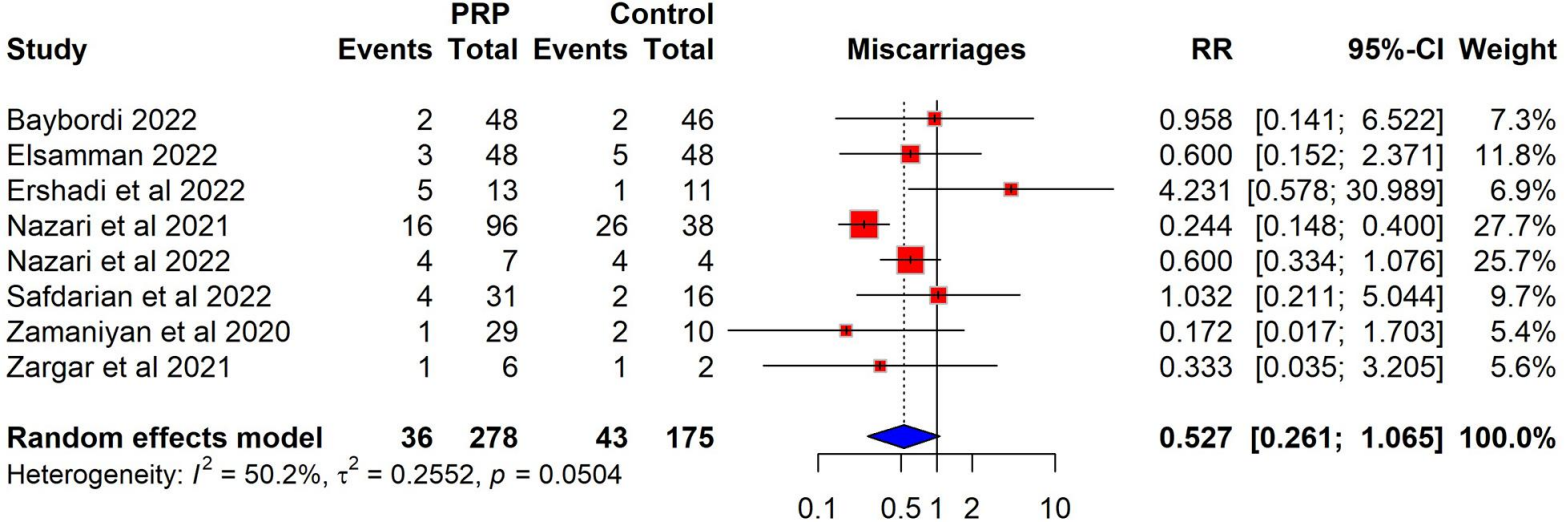
Pooled miscarriage rate following intrauterine platelet-rich plasma (PRP) infusion in women with unexplained recurrent implantation failure (RIF).

Only eight studies reported a miscarriage rate. The pooled results reported that the administration of PRP did not significantly affect miscarriage rate (RR: 0.52, CI: 0.26–1.06, *I*^2^ = 50.2%) (Figure 6). The quality of evidence was very low, and there was likely overestimation of the effect.

#### Quality assessment

The methodological quality of the included systematic reviews was determined using the AMSTAR checklist. The evaluation of the 15 studies on PRP in RIF treatment revealed a mixed quality profile. Four studies were rated as high quality, demonstrating robust methodology with scores of nine or higher (no critical weakness) (4, 6, 19, 20), while 10 studies fell into the moderate category (5–8), showing adequate but inconsistent adherence to criteria such as *a priori* design and publication bias assessment (Table 3). Of the 15 included systematic reviews, GRADE was reported by only five (4, 6, 10, 16, 19). These systematic reviews have reported GRADE as very low to moderate evidence for the outcomes included (Table 1).

**Table 3:** AMSTAR checklist for quality assessment of included reviews.

AMSTAR checklist questions	Included systematic reviews														
	Anitua et al. (19)	Busnelli et al. (10)	Deng et al. (11)	Huang et al. (13)	Kaur et al. (6)	Hu et al. (12)	Liu et al. (14)	Li et al. (5)	Ma and Pu (15)	Soliman et al. (18)	Panda et al. (16)	Shalma et al. (17)	Elsalam Elgendy et al. (21)	Maleki-Hagiagha et al. (20)	Maged et al. (4)
1. Was an “*a priori*” design provided?	Yes	No	Yes	Yes	Yes	No	Yes	Yes	Yes	Yes	Yes	No	No	Yes	Yes
2. Was there duplicate study selection and data extraction?	Yes	Yes	Yes	Unclear	Yes	Unclear	Yes	Yes	Yes	Yes	Yes	Unclear	Unclear	Yes	Yes
3. Was a comprehensive literature search performed?	Yes	Yes	Yes	Yes	Yes	Yes	No	Yes	Yes	Yes	Yes	Yes	No	Yes	Yes
4. Was the status of publication (i.e., grey literature) used as an inclusion criterion?	No	No	No	No	No	No	No	No	No	No	No	No	No	No	Yes
5. Was a list of studies (included and excluded) provided?	Yes	Yes	Yes	No	No	Included-Yes; Excluded—No	Yes	Yes	Yes	No	No	Included-Yes; Excluded—No	Included-Yes; Excluded—No	Yes	Included-Yes; Excluded—No
6. Were the characteristics of the included studies provided?	Yes	Yes	Yes	Yes	Yes	Yes	Yes	Yes	Yes	Yes	Yes	Yes	Yes	Yes	Yes
7. Was the scientific quality of the included studies assessed and documented?	Yes	Yes	Yes	Yes	Yes	Yes	Yes	Yes	Yes	Yes	Yes	Yes	Yes	Yes	Yes
8. Was the scientific quality of the included studies used appropriately in formulating conclusions?	Yes	Yes	No	Unclear	Yes	Yes	Unclear	Unclear	Unclear	Unclear	Yes	Yes	No	Unclear	Yes
9. Were the methods used to combine the findings of studies appropriate?	Yes	Yes	Yes	Yes	Yes	Yes	Yes	Yes	Yes	Yes	Yes	Yes	Yes	Yes	Yes
10. Was the likelihood of publication bias assessed?	No	Yes	Yes	No	Yes	No	No	Yes	Yes	No	No	No	No	Yes	Yes
11. Was the conflict of interest included?	Yes	No	No	Yes	Yes	Yes	Yes	Unclear	Yes	Unclear	Yes	Yes	Yes	Yes	Yes
AMSTAR Score	**High**	**Moderate**	**Moderate**	**Moderate**	**High**	**Moderate**	**Moderate**	**Moderate**	**Moderate**	**Moderate**	**Moderate**	**Moderate**	**Moderate**	**High**	**High**

#### Publication bias

**Figure 7:**
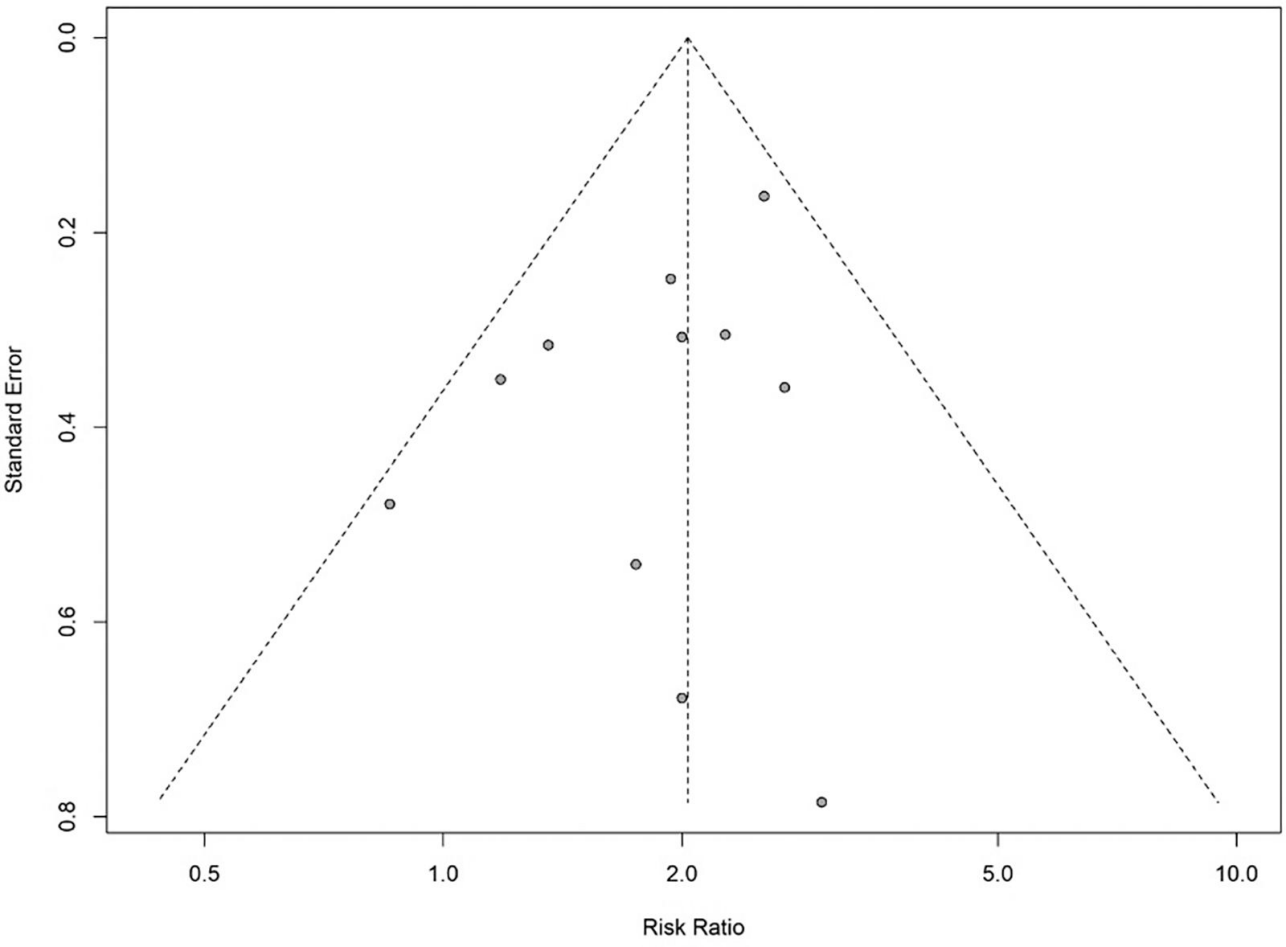
Funnel plot assessing publication bias for clinical pregnancy rate following intrauterine PRP infusion in women with unexplained recurrent implantation failure (RIF). The funnel plot displays the relationship between the effect size (clinical pregnancy rate) and its standard error across the included meta-analyses. Each dot represents an individual study or comparison. Symmetry of the plot suggests low risk of publication bias, whereas asymmetry may indicate potential bias or heterogeneity. Vertical line denotes the pooled effect estimate.

**Figure 8:**
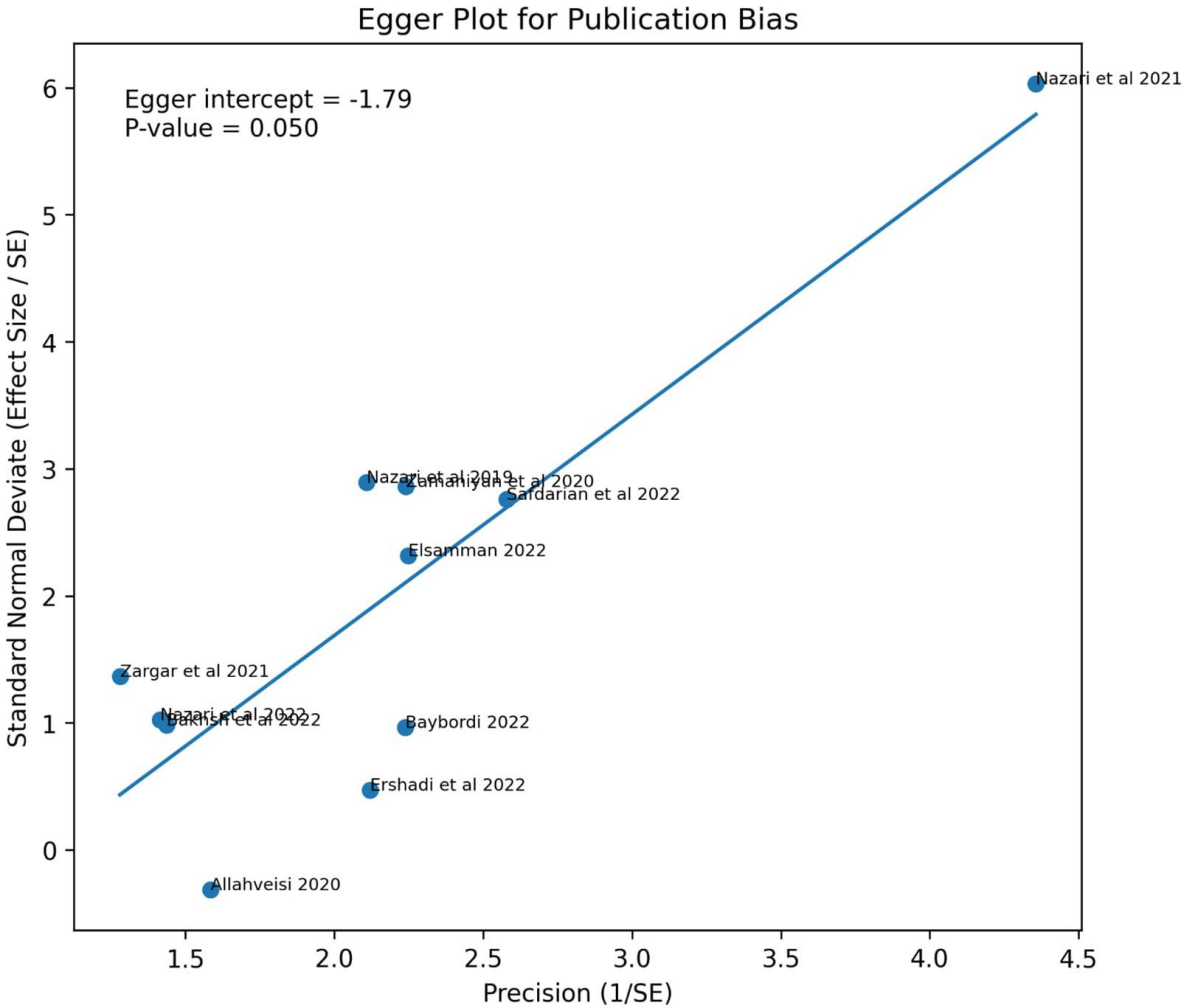
Egger plot depicting publication bias.

The funnel plot showed the studies to have low publication bias (Figure 7). Further Egger's regression test demonstrated borderline evidence of funnel plot asymmetry (intercept = −1.79, *p* = 0.050), suggesting the possibility of small-study effects or publication bias. However, interpretation should be cautious, given the limited number of included studies (Figure 8).

## Discussion

### Main findings

PRP, an autologous concentrate derived from a patient's blood, is rich in platelets, which release a wide range of growth factors and cytokines upon activation. These bioactive molecules play crucial roles in repairing and regenerating tissues, making PRP an attractive therapeutic option for enhancing endometrial receptivity (2, 36). One of the primary mechanisms is the enhancement of endometrial regeneration. Growth factors such as PDGF, VEGF, and transforming growth factor-beta (TGF-β) stimulate proliferation and migration of endometrial cells, promoting tissue repair (37). PRP also encourages thickening of the endometrial lining, an important determinant of implantation success. Another key mechanism involves the promotion of angiogenesis (38). EGF and other angiogenic mediators in PRP stimulate the formation of new blood vessels, improving uterine blood flow and oxygen delivery to the endometrium. This enhanced vascularisation supports embryo implantation and early placental development. PRP also exerts immunomodulatory and anti-inflammatory effects (39). By releasing anti-inflammatory cytokines, it creates a favourable immune environment that reduces chronic endometrial inflammation and supports embryo acceptance. In addition, PRP influences the activity of local immune cells, contributing to a more receptive uterine milieu. Finally, PRP may stimulate endometrial stem and progenitor cells and support extracellular matrix remodelling (36). These actions restore normal endometrial architecture and functionality, further improving receptivity. Through these multifaceted mechanisms, PRP creates a biologically favourable environment for embryo implantation, offering potential benefits to women with RIF who have not responded to conventional therapies.

#### Interpretation

This umbrella review consolidates findings from existing systematic reviews and meta-analyses on the use of PRP in managing recurrent implantation failure. A comprehensive literature search revealed a total of 15 systematic reviews addressing the research question of the role of PRP in RIF. There was significant overlap of studies reported in various reviews, as shown by a high CCA index of 42%. This indicates that many of the reviews synthesise data from the same primary studies, which reduces the independence of evidence and may limit the robustness of conclusions. Very high overlap also indicates that the overall results of the systematic reviews cannot be compiled into a meta-analysis. Although the qualitative review of these reviews has shown a beneficial effect of PRP in RIF, this result cannot be recommended with certainty, as GRADE analysis was performed in only five (4, 6, 10, 16, 19) of the 15 studies. Meta-analysis of the individual outcomes, after extracting data from all studies, also revealed an overall beneficial effect of PRP in terms of various outcomes, i.e., live birth rate, clinical pregnancy rate, and implantation. However, this may be an overestimation of the true effect due to the low quality of evidence or the quality of evidence not being assessed. In addition, not all outcomes were reported in each study within the reviews, e.g., live birth rate has been reported in only six trials (23, 24, 27, 28, 30, 35) and clinical pregnancy rate in 11 trials 2024 (23–32, 35).

To avoid any bias, a systematic review must be prospectively registered with a clear protocol. The methodological quality of the included reviews varied, with only 11 providing an *a priori* design, while the remaining four lacked this critical element. The absence of a pre-specified protocol in these reviews increases the potential for bias, selective reporting, and data-driven conclusions, thereby reducing confidence in their findings and slightly lowering the overall certainty of evidence in this umbrella review.

Safety data for PRP are reassuring, as it is an autologous product with minimal immunogenicity and a very low risk of infection or adverse reactions (40). However, long-term safety data, especially regarding perinatal and neonatal outcomes, are sparse and warrant further investigation. Moreover, head-to-head comparisons with other adjuvant therapies, such as granulocyte colony-stimulating factor or endometrial scratching, are lacking, limiting the understanding of PRP's relative effectiveness. Given these limitations, PRP should currently be considered an experimental therapy for RIF. Its use in clinical practice should be reserved for selected patients and preferably within the context of research protocols. Future studies should focus on multicentre randomised controlled trials with adequate sample sizes, uniform definitions of RIF, and standardised PRP preparation and administration protocols. In addition, mechanistic studies are warranted to clarify the pathways through which PRP exerts its effects on endometrial receptivity.

Despite promising findings, the overall quality of evidence remains low to moderate. Many included studies are small-scale, single-centre trials with methodological limitations and a high risk of bias. Heterogeneity of protocols is a major challenge in interpreting the results. There is considerable heterogeneity in the definition of RIF across studies, as well as in PRP preparation methods, (single vs. double spin techniques), concentrations, and administration protocols, which complicates the interpretation of results. Standardisation in these areas is crucial to strengthen the evidence base.

Although the GRADE assessment is an important component of evidence synthesis, in the present umbrella review, *de novo* meta-analysis was based on data derived from previously published systematic reviews rather than primary studies. Due to the lack of access to consistent, study-level data (e.g., detailed risk of bias domains, inconsistency, and imprecision across individual trials), a robust and reliable GRADE assessment for the pooled outcomes could not be performed.

The novelty of our review lies in its narrow RIF focus, the overlap analysis conducted, and the *de novo* re-analysis of the primary trials.

### Strengths and limitations

#### Strengths

This umbrella review compiles the literature on the use of PRP in RIF, as most of the systematic reviews have used small sample sizes. Adherence to the JBI framework and PRISMA guidelines ensures rigorous methodology, and the AMSTAR criteria was used to assess the methodological quality of included reviews. The Cochrane tool for ROB assessment and GRADE assessment ensure rigorous evidence evaluation. We have included systematic reviews with only RCTs to minimise heterogeneity.

#### Limitations

Given that this review is based on published data, the weaknesses and biases inherent to original reviews might affect the final results. In addition, some observational studies might be missing from the literature, as we have included only systematic reviews with RCTs.

## Conclusion

PRP appears to be a promising therapeutic option for women with recurrent implantation failure. However, the current evidence is limited by very high overlap, methodological heterogeneity, and reliance on small, low-quality trials. Until robust, high-quality evidence emerges, the use of PRP should be approached with caution and ideally confined to research settings.

### Implications for future research

While PRP is a promising, low-risk intervention that may benefit selected women with RIF, particularly those with thin endometrium or poor endometrial receptivity, current evidence is insufficient to support its routine use in clinical practice. Clinicians should consider PRP within the context of clinical trials or as an individualised therapy after thorough counselling regarding the limited certainty of benefits. Future investigations should focus on large, multicentre randomised controlled trials using standardised PRP preparation and administration protocols, as well as uniform definitions of RIF. Mechanistic studies are warranted to further elucidate the molecular pathways through which PRP enhances endometrial receptivity. In addition, long-term maternal and neonatal outcomes, cost-effectiveness analyses, and head-to-head comparisons with other adjuvant therapies are warranted.

## Data Availability

The original contributions presented in the study are included in the article/Supplementary Material, further inquiries can be directed to the corresponding author.
