# Supplementary material for: Role of platelet-rich plasma in unexplained recurrent implantation failure: an umbrella review
Source: Front Reprod Health. 2026 Jul 8;8:1856964. doi: 10.3389/frph.2026.1856964 (PMC13388886; doi:10.3389/frph.2026.1856964)
Supplement: Supplementary file 1 [file Table1.docx]

**Supplementary Table: Details of Search Strategy**

**Search strategy**

| **Database** | **Search Query** | **Results** |
| --- | --- | --- |
| PubMed | ("PRP"[All Fields] OR "Platelet Rich Plasma"[All Fields]) AND ("RIF"[All Fields] OR "recurrent Implantation failure"[All Fields]) AND ("Systematic review"[All Fields] OR "Meta-analysis"[All Fields] OR "Meta-analysis"[All Fields]) | 14 |
| EMBASE | ('prp' OR 'platelet rich plasma') AND ('rif' OR 'recurrent implantation failure') AND ('systematic review' OR 'meta-analysis' OR 'meta analysis') | 21 |
| Cochrane | ('prp' OR 'platelet rich plasma') AND ('rif' OR 'recurrent implantation failure') in All Text - (Word variations have been searched) | 6 |
| Web of Science | ('prp' OR 'platelet rich plasma') AND ('rif' OR 'recurrent implantation failure') AND ('systematic review' OR 'meta-analysis' OR 'meta analysis') (All Fields) | 16 |
