# Supplementary material for: Role of platelet-rich plasma in unexplained recurrent implantation failure: an umbrella review
Source: Front Reprod Health. 2026 Jul 8;8:1856964. doi: 10.3389/frph.2026.1856964 (PMC13388886; doi:10.3389/frph.2026.1856964)
Supplement: Supplementary file 2 [file Table2.docx]

| **Author, Year** | **Country** | **Duration** | **Participants (Number; Age; BMI)** | **Inclusion criteria** | **Exclusion criteria** | **Intervention** | **Control** | **Embryo transfer** | **Clinical pregnancy rate (PRP vs Control; RR [95% CI])** | **Biochemical pregnancy rate (PRP vs Control; RR [95% CI])** | **Implantation rate (PRP vs Control; RR [95% CI])** | **Live birth rate (PRP vs Control; RR [95% CI])** | **Miscarriage rate (PRP vs Control; RR [95% CI])** |
| --- | --- | --- | --- | --- | --- | --- | --- | --- | --- | --- | --- | --- | --- |
| Bakhsh et al. 2022 | Iran | 2020 | 100; <40y; BMI <30 | RIF with ≥4 embryos | Medical/genetic/uterine disorders | PRP 0.5 mL 48h before ET | Control | Frozen | 6/50 vs 3/50; RR 2.00 (0.53–7.56) | NR | NR | NR | NR |
| Ershadi et al. 2022 | Iran | 2019 | 90; <40y; BMI <30 | 2–3 IVF failures | Uterine/systemic disorders | PRP 0.5 mL | Control | Frozen D3 | 13/45 vs 11/45; RR 1.18 (0.59–2.35) | 16/45 vs 12/45; RR 1.33 (0.71–2.49) | NR | NR | 5/13 vs 1/11; RR 4.23 (0.58–30.99) |
| Nazari et al. 2019 | Iran | 2016–2017 | 97; <40y; BMI <30 | RIF | Uterine/genetic disorders | PRP 0.5 mL | Control | Frozen | 22/49 vs 8/48; RR 2.69 (1.33–5.45) | 26/49 vs 13/48; RR 1.96 (1.15–3.34) | NR | NR | NR |
| Nazari et al. 2021 | Iran | 2018–2020 | 393; 18–38y; BMI ≤30 | RIF ≥3 failures | Hormonal/PCOS/endometriosis | PRP 0.5 mL | Control | Frozen | 96/196 vs 38/197; RR 2.54 (1.85–3.50) | 101/196 vs 49/197; RR 2.07 (1.57–2.74) | NR | 77/196 vs 11/197; RR 7.04 (3.86–12.82) | 16/96 vs 26/38; RR 0.24 (0.15–0.40) |
| Nazari et al. 2022 | Iran | 2019–2020 | 40; <40y | Recurrent pregnancy loss | Genetic/immunologic disorders | PRP 0.5 mL | Control | Fresh | 7/20 vs 4/20; RR 1.75 (0.61–5.05) | NR | NR | 3/20 vs 0/20; RR 7.00 (0.39–127.13) | 4/7 vs 4/4; RR 0.60 (0.33–1.08) |
| Rageh et al. 2020 | Bahrain | 2018–2019 | 150; <40y | RIF | Systemic/genetic disorders | PRP 1 mL | Control | Fresh | NR | 32/75 vs 11/75; RR 2.91 (1.59–5.33) | NR | NR | NR |
| Safdarian et al. 2022 | Iran | 2017–2020 | 120; 20–40y | RIF ≥3 failures | Genetic/hormonal disorders | PRP 0.5 mL | Control | Frozen | 31/60 vs 16/60; RR 1.94 (1.19–3.15) | 31/60 vs 18/60; RR 1.72 (1.09–2.72) | 20/60 vs 13/60; RR 1.54 (0.85–2.80) | 35/60 vs 17/60; RR 2.06 (1.31–3.25) | 4/31 vs 2/16; RR 1.03 (0.21–5.04) |
| Zamaniyan et al. 2020 | Iran | 2016–2019 | 98; 20–40y | RIF | Hematologic/genetic disorders | PRP 0.5 mL | Control | Frozen | 29/55 vs 10/43; RR 2.27 (1.25–4.12) | 20/55 vs 10/43; RR 1.56 (0.82–2.98) | 35/55 vs 15/43; RR 1.82 (1.16–2.87) | NR | 1/29 vs 2/10; RR 0.17 (0.02–1.70) |
| Zargar et al. 2021 | Iran | 2018 | 80; <40y | RIF ≥2 failures | Uterine/genetic disorders | PRP 1.5 mL | Control | Frozen | 6/40 vs 2/40; RR 3.00 (0.64–13.98) | NR | NR | 5/40 vs 0/40; RR 11.00 (0.63–192.48) | 1/6 vs 1/2; RR 0.33 (0.04–3.21) |
| Allahveisi et al. 2020 | Iran | 2018–2019 | 50; <40y | RIF | NR | PRP 0.5 mL | Ringer | Frozen | 6/25 vs 7/25; RR 0.86 (0.34–2.19) | NR | NR | 6/25 vs 7/25; RR 0.86 (0.34–2.19) | NR |
| Elsamman et al. 2022 | Egypt | NR | 96; 18–35y | RIF + thin endometrium | Comorbidities | PRP 0.5 mL | Control | Fresh/Frozen | 22/48 vs 11/48; RR 2.00 (1.09–3.66) | 25/48 vs 16/48; RR 1.56 (0.96–2.53) | 59/101 vs 30/87; RR 1.69 (1.21–2.36) | NR | 3/48 vs 5/48; RR 0.60 (0.15–2.37) |
| Obidniak et al. 2017 | Russia | NR | 90; 28–39y | RIF | NR | PRP 2 mL | Control | NR | NR | NR | 18/45 vs 9/45; RR 2.00 (1.01–3.97) | NR | NR |
| Baybordi et al. 2022 | Iran | 2017–2019 | 94 | RIF | NR | PRP 0.5–1 mL | Control | Blastocyst | 17/48 vs 12/46; RR 1.36 (0.73–2.52) | 21/48 vs 12/46; RR 1.68 (0.94–3.00) | NR | 9/48 vs 8/46; RR 1.08 (0.46–2.55) | 2/48 vs 2/46; RR 0.96 (0.14–6.52) |
| Dawood et al. 2022 | Egypt | 2018–2021 | 104; 20–35y | RIF | NR | PRP | Control | Blastocyst | NR | NR | NR | NR | NR |

**Supplementary Table 2: Primary study Contribution to pooled outcomes**
